# Amplified drought induced by climate change reduces seedling emergence and increases seedling mortality for two Mediterranean perennial herbs

**DOI:** 10.1002/ece3.8295

**Published:** 2021-11-10

**Authors:** Suzon Garnier, Emma Giordanengo, Arne Saatkamp, Mathieu Santonja, Ilja M. Reiter, Jean‐Philippe Orts, Thierry Gauquelin, Eric Meineri

**Affiliations:** ^1^ Aix Marseille Univ, Avignon Univ CNRS, IRD, IMBE Marseille France; ^2^ CNRS, ECCOREV Aix‐en‐Provence France

**Keywords:** aridification, climate change, emergence, mortality, *Silene italica*, *Silene nutans*

## Abstract

Seedling recruitment is a bottleneck for population dynamics and range shift. The vital rates linked to recruitment by seed are impacted by amplified drought induced by climate change. In the Mediterranean region, autumn and winter seedling emergence and mortality may have strong impact on the overall seedling recruitment. However, studies focusing on the temporal dynamic of recruitment during these seasons are rare. This study was performed in a deciduous Mediterranean oak forest located in southern France and quantifies the impact of amplified drought conditions on autumn and winter seedling emergence and seedling mortality rates of two herbaceous plant species with meso‐Mediterranean and supra‐Mediterranean distribution (respectively, *Silene italica* and *Silene nutans*). Seedlings were followed from October 2019 to May 2020 in both undisturbed and disturbed plots where the litter and the aboveground biomass have been removed to create open microsites. Amplified drought conditions reduced seedling emergence and increased seedling mortality for both *Silene* species but these negative effects were dependent on soil disturbance conditions. Emergence of *S. italica* decreased only in undisturbed plots (−7%) whereas emergence of *S*. *nutans* decreased only in disturbed plots (−10%) under amplified drought conditions. The seedling mortality rate of *S*. *italica* was 51% higher under amplified drought conditions in undisturbed plots while that of *S*. *nutans* was 38% higher in disturbed plots. Aridification due to lower precipitation in the Mediterranean region will negatively impact the seedling recruitment of these two *Silene* species. Climate change effects on early vital rates may likely have major negative impacts on the overall population dynamic.

## Graphical abstract

This study was performed in a deciduous Mediterranean oak forest and quantifies the impact of amplified drought conditions on autumn and winter seedling emergence and seedling mortality rates of two *Silene* species. Amplified drought conditions reduced seedling emergence and increased seedling mortality for both *Silene* species but these negative effects were dependent on soil properties.

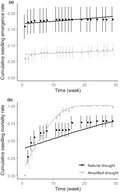

## 1 INTRODUCTION

The Mediterranean basin is one of 34 biodiversity hotspots and englobes a particularly high plant species richness with 25,000 to 30,000 species (Myers et al., 2000), of which 44% have restricted distribution ranges (Gimenez‐Benavides et al., 2007; Médail & Diadema, 2009; Médail & Verlaque, 1997). This region is also a climate change hotspot, where climate change is particularly severe (Giorgi, 2006). Climate models predict an increase in temperature and a reduction in rainfall, resulting in an extended drought period (Christensen et al., 2013; de Luis et al., 2010). Since water is already one of the main limiting factors for the Mediterranean vegetation (Penuelas et al., 2004), further amplified drought may have strong impacts on population dynamics, especially for populations located near their range limits (Hampe & Petit, 2005).

Population dynamics rely on the success of recruitment, which requires the dispersal of viable seeds followed by the successful establishment of seedlings (Frei et al., 2018). Seedling emergence and seedling survival are two important bottlenecks for recruitment (Gimenez‐Benavides et al., 2008; Graae et al., 2011). Furthermore, low survival rate of seedlings has been shown to have a particularly strong impact on population dynamics (Gómez‐Aparicio et al., 2008; Zeiter et al., 2006). Both seedling emergence and seedling survival generally respond negatively to amplified drought (Reviewed in Walck et al., 2011). However, the effects of amplified drought on recruitment might be less negative in Mediterranean regions because major seedling recruitment events take place during the autumn (Baskin & Baskin, 1998; Levine et al., 2011; Merritt et al., 2007) when the temperature is not so hot anymore. Although such counterintuitive effects seem uncommon for seedling emergence (Matías et al., 2018), seedling survival may not be affected or may even be promoted by amplified drought in the Mediterranean region (del Cacho et al., 2013). To better understand how expected amplified drought will impact seedling emergence and seedling survival in the Mediterranean region, we need to monitor seedling cohorts during the cold seasons. Yet, such studies are still uncommon.

In vitro studies show considerable variations in responses of seedling emergence and survival to climatic changes among study systems and species (Carta, 2016; Fernández‐Pascual et al., 2013; Merritt & Dixon, 2011). A cue to better understand variation in reactions may come from the relative positions in distributional ranges. For instance, Gerst et al. (2011) showed that increased variability of fecundity and survival in desert annuals is related to their position in geographical distributional areas. However, other studies carried out in colder regions found that seedling recruitment responses to soil moisture were not linked to the positions of the experimental plots within species ecological niches (Meineri et al., 2013; Töpper et al., 2018). We find it hence particularly interesting to study the effects of increased autumn drought on both seed germination and seedling survival for plants *in situ* for two congeneric species at their upper and lower distributional limit.

*In situ* studies investigating the impact of amplified drought on seedling recruitment are often based on reciprocal transplants of seeds or seedlings (Giménez‐Benavides et al., 2008; Meineri et al., 2013; Töpper et al., 2018) or on seed sowing experiments with a watering treatment (Kimball et al., 2010; Larson et al., 2015; O'Brien, Ong, et al., 2017). However, reciprocal transplant experiments do not separate the effects of humidity from other potential collinear factors that may vary in parallel to natural humidity gradients (Meineri et al., 2013). Particularly interesting in this context are field experiments excluding rainfall (e.g., del Cacho et al., 2013), because they can more effectively mimic variations in precipitation and increasing drought duration (O’Brien, Reynolds, et al., 2017; Polade et al., 2017). Studies using rainfall manipulation to investigate seedling emergence and survival under changing climate (del Cacho et al., 2013; Classen et al., 2010) have shown the positive role of soil moisture for emergence and the importance of timing and frequency of rainfall for seedling emergence and survival. Excluding the first relevant rainfall event and monitoring timing of seedling emergence and survival during the onset of moist season in water‐limited ecosystems might here be key to understand variability between species.

In this study, we experimentally manipulated precipitation patterns (by extending the summer drought period) in a deciduous Mediterranean oak forest located in southern France and quantify the impact of amplified drought on seedling emergence and seedling mortality of two species at their climatic range limits. These species are *Silene italica*, a forb species with meso‐Mediterranean distribution, reaching its colder range limit at the experimental site and *Silene nutans*, a forb species with supra‐Mediterranean distribution, reaching its warmer range limit at the experimental site. We carried out a seed sowing experiment under natural and amplified drought conditions created *via* rain exclusion, and we monitored seedling emergence and mortality over fall, winter, and spring. Based on the literature, we hypothesized that amplified drought reduces (a) seedling recruitment rates and (b) increases seedling mortality of *Silene nutans* (meso‐Mediterranean distribution) but (c) will have no effect on both early vital rates for *Silene italica* (supra‐Mediterranean distribution).

## 2 MATERIALS AND METHODS

### 2.1 Study site

This study was conducted on the experimental site O_3_HP (“Oak Observatory at the Observatoire de Haute Provence”), part of the research infrastructure AnaEE‐France, located in the Luberon Natural Regional Park (43°45′ 34.26″ N; 5◦17′57.84″ E), Southern France. The study site is a deciduous oak forest where Downy oak (*Quercus pubescens* Willd) and Montpellier maple (*Acer monspessulanum* L.) are the two dominant tree species (see Santonja et al., 2015 for further details). The shrub layer is dominated by *Cotinus coggygria*, *Genista hispanica*, *Cornus mas*, *Cytisophyllum sessilifolium*, and *Amelanchier ovalis*, and the herbaceous layer is dominated by *Aphyllantes monspeliensis*, *Carex halleriana*, *Teucrium chamaedrys*, and *Melampyrum cristatum*. A dense litter layer of about 5 cm covers the ground during autumn and winter.

In order to simulate a drier climate, the study site is equipped with a rain exclusion device that dynamically excludes precipitations throughout the year since 2012 (see Santonja et al., 2015 for further details). Rain covers are deployed electronically on human demand via a web‐based interface, as to form a roof on a 15 m × 20 m steel construction above the 6 m high canopy, covering 300 m^2^ of ground. Water from exclusion is evacuated by gutters away from the site. An adjacent control parcel without device serves as control (i.e., subjected to ambient drought). The piloting scenario for precipitation exclusion closely simulates climate predictions. It is based on the relation of precipitation and temperature for hot years of local observations (1961–2010), which are extrapolated to +2°, respecting seasonality. As such, the usual one‐month summer drought period is increased from July to the end of September (i.e. Solomon et al., 2009) and the mean annual rainfall (829 mm) is targeted to 500–550 mm (corresponding to Giorgi & Lionello, 2008, −30 ± 10%) by mostly excluding whole rain events (e.g., Polade et al., 2014). In winter, the system is not manipulated below +4°C in order to avoid deterioration from snow loads, or mechanical strains due to freezing. On average since 2012, we have excluded 38 ± 5% of precipitation, and increased the annual number of dry days by 25 ± 8 days (Giorgi & Lionello, 2008; Guiot & Cramer, 2016; Polade et al., 2014). Volumetric soil water content (Hydra Probe II; Stevens Water Monitoring Systems, Inc., USA), hereafter simply referred to as “soil moisture”, is continuously monitored at 10 cm depth in the natural drought and amplified drought parcels with five sensors each. To standardize the individual response of the sensors, the data was scaled linearly to the span between a water‐saturated period (23.12.19) and a dry period (14.10.19), where the mean absolute readings of the ten sensors correspond to 0.41 l.l^−1^ and 0.13 l.l^−1^, respectively.

### 2.2 Model species

Two *Silene* species (Caryophyllaceae) were chosen to represent different positions in ecological niches: *Silene nutans* was selected as supra‐Mediterranean species. Its range extends from the northern Mediterranean region to southern Scandinavia, reaching its warmer (rear) edge at the experimental site (Hepper, 1956; Van Rossum et al., 2003). *Silene italica* has a meso‐Mediterranean distribution and spreads over the entire Mediterranean basin, reaching its colder (leading) edge at the experimental site (Du Pasquier & Jeanmonod, 2016). Both species are frequent in open mesic to dry woodlands and often grow in weakly disturbed habitats. These *Silene* are perennial plants, with reduced leaf canopies during cold and dry seasons. Seeds of these two species have generally a low dormancy and undergo a residual loss of dormancy upon exposure to hot summer temperatures (Arène et al., 2017; Walck et al., 2011). They germinate in autumn or winter (Baskin & Baskin, 1998). This pair of supra‐ and meso‐Mediterranean species was chosen because they are very close morphologically, have similar growing strategies, and are sufficiently frequent within and in the surrounding of the study site to enable seed collection.

### 2.3 Seed sowing experiment

We set up 15 experimental plots for each drought treatment (natural ND vs. amplified drought AD) in October 2019. The plots were chosen semi‐randomly, close to walking path to avoid disturbing the experimental site and avoiding places with large dense shrub cover in order to obtain similar light conditions for all experimental plots. Each plot measures 37 cm × 27 cm and contains eight sub‐plots of 5 cm × 5 cm. Previous studies have shown a very low rate of emergence in undisturbed communities (Larson et al., 2015). Therefore, the experiment was replicated in both undisturbed and disturbed plots where the litter and the aboveground biomass have been removed manually to create open microsites (Frei et al., 2018). For each species, we set up a pair of intact subplots and a pair of disturbed subplots. For each pair of subplots, one subplot received the seeds while the second subplot received no seed and served as a control to detect a possible emergence from the seed bank or natural seed rain (see Graae et al., 2011; Klanderud et al., 2017; Meineri et al., 2013 for similar set‐up). Thirty seeds were sown per subplot (apart from the seedbank control). The seeds of the two species were collected on the study site during summer 2019, cleaned and maintained dry at room temperature, and then sown in early October 2019 (week 41). The emergence censuses were carried out every week during the first month (October 2019), and then every two weeks until the end of the experiment, for a total of 20 reading dates between 2019/10/28 and 2020/05/15. During each survey, each new seedling identified was marked with a wooden toothpick to be able to record seedling mortality during the following weeks. For each survey and subplot, the number of seedlings recorded in the seedbank control subplot was subtracted from the seedling count of the corresponding experimental subplot. Emergence was however null in most plots.

**FIGURE 1:**
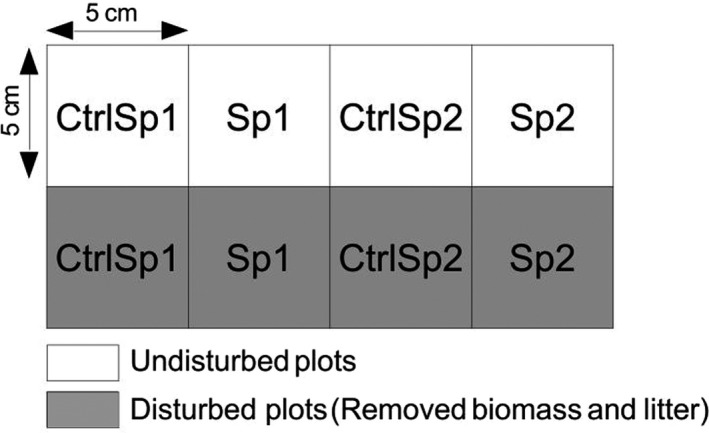
Set‐up of experimental plots. Fifteen experimental plots were installed within each of the two treatments (natural vs. amplified drought). Sp1: plot received 30 seeds of *Silene italica*; ctrlSp1: Seed‐bank control for *Silene italica*; Sp2: plot received 30 seeds of *Silene nutans*; ctrlSp1: Seed‐bank control for *Silene nutans*

The first major rain event occurred about two weeks after sowing and was excluded for the plots under amplified drought treatment. The rain exclusion device was then activated according to the piloting scenario described earlier (Figure 1).

### 2.4 Statistical analyses

Before any analysis on seedling recruitment, we accessed differences in soil moisture between the two drought treatments using a linear mixed effect model considering the date as a random variable.

For the two *Silene* species, we adjusted a set of logistic mixed effects models (GLMM with binomial error distribution), followed by post‐hoc Tukey HSD tests, to assess the effect of amplified drought on (a) the proportion of novel emerged seedlings recorded at each survey (hereafter referred to as weekly seedling emergence), (b) the cumulative proportion of emerged seedlings at each survey, and (c) the cumulative proportion of seedling mortality at each survey, and to investigate whether such effects differed between undisturbed and disturbed plots, cleaned from vegetation and litter. In these models, drought treatment (natural drought vs. amplified drought), soil disturbance (control vs. litter and vegetation removal), and their interaction were included as fixed explanatory variables. The survey date was included as a random variable to consider the repetition of surveys over time.

We then investigated the effects of amplified drought on weekly seedling emergence scores and searched if such effects differed between specific surveys. For both species, we fitted another set of logistic models (GLM with binomial error distribution), followed by post‐hoc Tukey HSD tests. This GLMs include the drought treatment, the sampling date, and their interactions as explanatory variables. These models specifically investigated whether the effect of amplified drought remains similar over the different surveys. To ease the interpretation, these models are based on data from either intact or disturbed plots, according to the results of the first set of models. For example, if the first set of models suggested that amplified drought impacts seedling emergence on disturbed plots only, we used only the data from disturbed plots for the subsequent analysis.

Likewise, we further investigate how the effect of amplified drought progressively varied over time for cumulative seedling emergence and cumulative mortality. For this, we fitted logistic mixed effect models including drought treatment, sampling date, and their interaction as fixed variables, this time using date as a continuous variable. Plot identity was included as a random variable.

For each of our analyses, overdispersion was checked by comparing residual deviance and residual degrees of freedom. When we detected over‐dispersion, we included a random variable identifying each observation (for GLMMs, Browne et al., 2005; Lawson, 1999) or we used quasibinomial distribution (for basic GLMs). A stepwise backward selection of variables was carried out for each model. All analyses were carried out in R (version 3.2.1, R Development Core Team, 2018). The “lmerTest” library was used to fit GLMMs (Kuznetsova et al., 2017) and the “emmeans” library for Tukey's tests (Lenth et al., 2018). The "RVAideMemoire" library was used to check out for the over‐dispersion in the GLMMs (Hervé & Hervé, 2020) and "ggplot2" was used to make the graphic representations (Wickham, 2016).

## 3 RESULTS

### 3.1 Soil moisture

**FIGURE 2:**
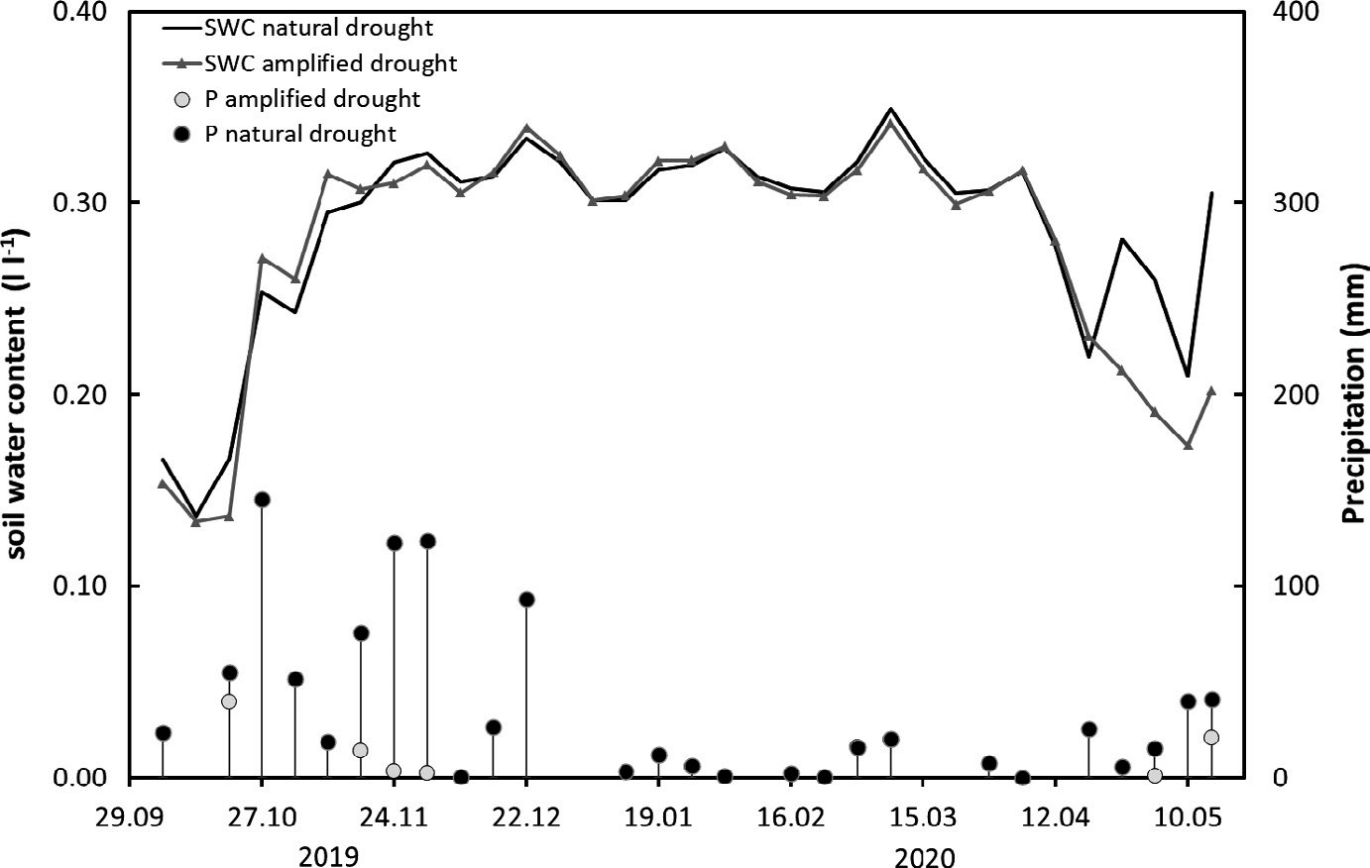
Weekly averages of soil water content (SWC) and the sum of precipitation (P, vertical bars)

Over the study period, soil moisture was lower in the amplified drought compared to the natural drought plots (*p* = .015). The excluded rainfall resulted in delayed soil moisture peaks under amplified drought plots in autumn 2019 and in a drastic decrease in soil moisture in spring 2020 (Figure 2). These periods match the functioning of the rain exclusion system, which is not deployed during the winter.

### 3.2 Seedling recruitment of *Silene italica*

For *S*. *italica*, the effect of amplified drought differed between disturbed and intact plots for weekly seedling emergence, cumulative seedling emergence, and cumulative seedling mortality rate (significant soil disturbance × drought interaction for the three models, Table 1, *p* < .001). We observed lower emergence rates (both weekly rate and cumulative rate) and higher seedling cumulative mortality rate with amplified drought in the intact plots only (Table 2, *p* < .001).

**TABLE 1:** Summary of mixed effect GLMs testing the overall effects of drought and soil disturbance treatments on seedling emergence, cumulative seedling emergence, and cumulative seedling mortality of *Silene italica* and *Silene nutans*

Species	Explanatory variable	Seedling emergence		Cumulative seedling emergence		Cumulative seedling mortality	
		Coefficient	*p*‐Value	Coefficient	*p*‐Value	Coefficient	*p*‐Value
*S. italica*	Intercept (natural drought)	−6.859	<.001	−1.790	<.001	0.565	.247
	Amplified drought	−0.821	<.001	−1.022	<.001	1.581	<.001
	Disturbance	0.886	<.001	0.908	<.001	0.203	.275
	Amplified drought: Disturbance	0.734	.002	0.937	<.001	−1.716	<.001
*S. nutans*	Intercept (natural drought)	−7.817	<.001	−3.510	<.001	1.394	<.001
	Amplified drought	−0.491	<.001	−0.182	.088	−1.01	.002
	Disturbance	1.853	<.001	2.052	<.001	−1.736	<.001
	Amplified drought: Disturbance	Removed	Removed	−0.431	<.001	2.144	<.001

“Removed”: removed during stepwise selection.

**TABLE 2:** Results of the post‐hoc Tukey tests performed on the logistic mixed models testing the overall effects of drought and soil disturbance treatments on seedling emergence rate, cumulative seedling emergence rate, and cumulative seedling mortality rate of *Silene italica* and *Silene nutans*

Species	Disturbance	Seedling emergence		Cumulative seedling emergence		Cumulative seedling mortality	
		Natural vs. amplified drought	*p*‐Value	Natural vs. amplified drought	*p*‐Value	Natural vs. amplified drought	*p*‐Value
*S. italica*	Undisturbed	0.821	<.001	1.022	<.001	−1.581	<.001
	Disturbed	0.086	.496	0.084	.331	0.135	.452
*S. nutans*	Undisturbed	–	–	0.182	.088	1.01	.003
	Disturbed	–	–	0.614	<.001	−1.13	<.001

“–”: Not tested because there was no effect of amplified drought

**FIGURE 3:**
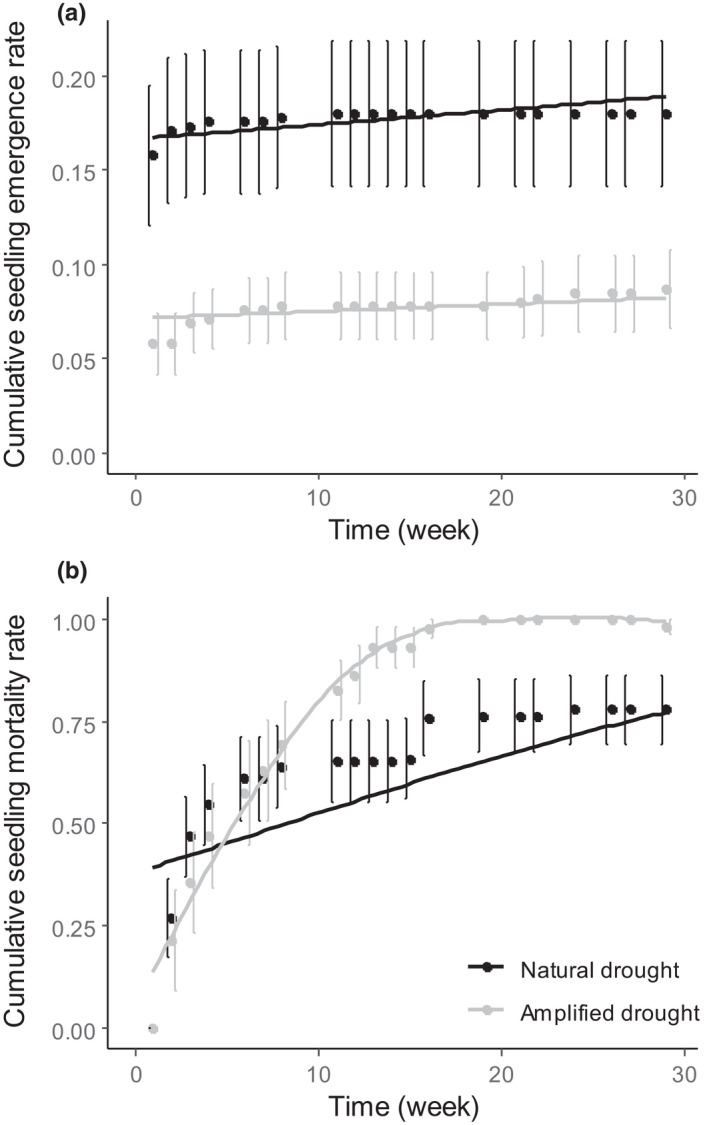
Cumulative seedling emergence rate (a) and cumulative seedling mortality rate (b) of *Silene italica* as a function of time (in number of weeks) from 2019/10/28 to 2020/05/14. Trend curves are issued from logistic models including time and drought treatment as explanatory variables (see methods and results for further details). These analyses consider intact plots only since no overall effect of amplified drought was found in plots where litter and vegetation were removed

Weekly seedling emergence rates over time showed a peak of emergence during the first week of the experiment (2019/10/28) followed by a rapid decline of seedling emergence. Accordingly, differences in weekly seedling emergence rates between natural and amplified drought were significant only for the first survey (*p* < .001), i.e., two weeks after sowing and a few days after the first excluded precipitation event. Cumulative emergence rate was lower with amplified drought (*p* < .001). This difference remained constant over the entire experimental period since no significant interaction between time and drought treatment was detected. We did not find any further effect of date, confirming that the number of emerged seedlings remained constant after the first peak in the first week and over the whole period (Figure 3a). At the end of the experiment (29th week, 2020/05/14), in the intact plot, about 19% of the seeds had emerged under natural drought condition while only 11% of the seeds had emerged under amplified drought condition.

Although seedling mortality was greater under natural drought during the first few weeks, a significant time ×drought interaction (*p* < .001, Table 3) indicated that this trend rapidly reversed (Figure 3b). Indeed, in intact plots, seedling mortality rate increased faster over time under amplified drought and became higher compared to natural drought at the end of the experiment (100% vs. 75% for amplified and natural drought conditions, respectively).

**TABLE 3:** Logistic models (GLM with binomial error distribution) investigating the effect of amplified drought condition on seedling cumulative emergence and mortality rate over time. Models are presented for both intact and disturbed plots (removal of vegetation and litter) for *S*. *nutans* as the amplified drought condition impacted emergence on both types of plot

Species	Disturbance	Explanatory variable	Cumulative seedling emergence rate		Cumulative seedling mortality rate	
			Coefficient	*p*‐Value	Coefficient	*p*‐Value
*S. italica*	Intact	Intercept (natural drought)	−1.534	<.001	−0.488	.003
		Time	Removed	Removed	0.059	<.001
		Amplified drought	0.956	<.001	−1.431	.001
		Time: Amplified drought	Removed	Removed	0.277	<.001
*S. nutans*	Disturbed	Intercept (natural drought)	−1.726	<.001	−1.492	<.001
		Time	0.029	<.001	0.075	<.001
		Amplified drought	−0.563	<.001	0.946	<.001
		Time: Amplified drought	Removed	Removed	Removed	Removed
	Intact	Intercept (natural drought)	–	–	0.668	.032
		Time	–	–	0.020	.272
		Amplified drought	–	–	−1.913	<.001
		Time: Amplified drought	–	–	0.103	<.001

“Removed”: removed during stepwise selection; “–”: Not tested because there was no overall effect of amplified drought.

### 3.3 Seedling recruitment of *Silene nutans*

Over all surveys, amplified drought had a negative effect on the seedling emergence in both intact and disturbed plots (*p* < .001, Table 1). However, this negative effect only appeared in disturbed plots for cumulative seedling emergence (significant drought × disturbance interaction, *p* < .001, Tables 1 and 3).

**FIGURE 4:**
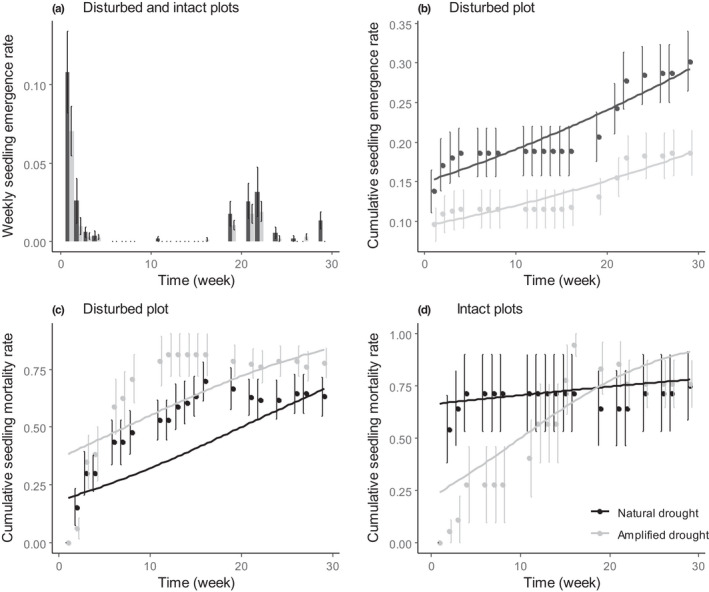
Seedling emergence rate (a), cumulative seedling emergence rate (b), and cumulative seedling mortality rate (c and d) of *Silene nutans* as a function of time (in number of weeks) from 2019–10–28 to 2020–05–14. Trend curves are issued from logistic mixed effect models (see methods and results for further details)

As for *S*. *italica*, a peak of seedling emergence appeared in the first week (2019/10/28) followed by a rapid decrease in seedling emergence (Figure 4a). However, for *S*. *nutans*, the recruitment started again in the following spring (24th week after the start of the experiment, 2020/04/09). The effect of the amplified drought did not differ between surveys as we found no significant interaction between survey date and drought treatment for both weekly and cumulative seedling emergence rates (Table 3, Figure 4a). At the end of the experiment, 22% of the seeds had emerged under natural drought while only 15% of them emerged under amplified drought condition, considering both disturbed and intact plots (Figure 4a,b).

Seedling mortality rate responded differently to drought in undisturbed and disturbed plots (significant drought × soil disturbance interaction, *p* < .001, Table 1). There was a higher mortality under amplified drought in disturbed plots all along the monitoring period (+38%, *p* < .001, Table 2). In the intact plots, the model suggests lower mortality under amplified drought at the beginning of the experiment (−57%, *p* < .001, Table 2) (Figure 4c,d). However, the significant drought × time interaction in intact plots suggests that this lower mortality under amplified drought reversed by the 15th week (February 2020), meaning higher mortality rates under amplified drought from that day on (*p* < .001, Table 3, Figure 4d). At the end of the experiment, seedling mortality was 25% higher under amplified drought condition.

## 4 DISCUSSION

### 4.1 Drought impact on seedling emergence

Overall, the amplified drought treatment reduced significantly soil moisture, although it was only visible after excluded rainfall events during the autumn and the spring (see Figure 2). For both species, *Silene italica* and *Silene nutans*, lower rates of seedling emergence were recorded under amplified drought. The strength and timing of these effects differed between species and whether vegetation and litter had been removed. Indeed, seedling emergence of *S*. *italica* was significantly lower under amplified drought treatment only in intact plots, whereas for *S*. *nutans*, the effects of amplified drought were only observed on disturbed plots.

Greater recruitment in open microsites compared to intact vegetation has been reported frequently (Eriksson & Froborg, 1996; Frei et al., 2018). The different responses to abiotic stress may be linked to the location of our experimental study site within the distribution range of the two species. *S*. *nutans* is at its warm range limit at the study site, where the stress gradient hypothesis predicts a higher importance of competition (Brooker, 2006a; Klanderud et al., 2017, 2021). For these species, a release of competition might be necessary to obtain sufficient seedling recruitment to detect an effect of amplified drought. In contrast, for *S*. *italica*, the study site is at the cold range limit where facilitation processes are predicted to play a major role, among other reasons, because dense vegetation may create a more favorable warmer microclimate (Brooker, 2006b; Klanderud et al., 2017, 2021).

A peak characterizes the seedling emergence of both species during autumn, two weeks after sowing, and a few days after the first rainfall event that was excluded under amplified drought treatment. Seedling emergence then almost stopped during winter. While the difference in seedling emergence between the two drought treatments only appeared during the peak of emergence, its impact on the cohorts was maintained throughout the experiment. For *S*. *nutans*, seedling emergence restarted in spring and the effects of amplified drought increased again. Thus, the exclusion of one or two rainfall events has tremendous impacts on the final number of emerging seedlings, implying a long‐term negative impact on population dynamics. Such long periods of aridity are what is predicted in the near future (Christensen et al., 2013; Penuelas et al., 2004), and will certainly lower recruitment rates for many species.

Indeed, lower seedling emergence rates under drier conditions have been observed for many species and in many ecosystems (Walck et al., 2011). However, we found this negative effect regardless of whether populations are at their warmer or colder distributional limit. Several authors suggest that seedling recruitment is limited by low emergence rates (Giménez‐Benavides et al., 2011; O’Brien, Reynolds, et al., 2017; Sternberg et al., 1999). In the Mediterranean region where conditions are already dry and where climate projections predict greater warming rates together with lower precipitation, the impact of lower seedling emergence on population dynamics is likely to be even greater (Giménez‐Benavides et al., 2011).

### 4.2 Drought impact on seedling mortality

The results show a negative effect of amplified drought on seedling survival for both *Silene* species. Results differ according to species and disturbance treatment with a significant effect of amplified drought in both disturbed and undisturbed plots for *S*. *nutans*, but only in undisturbed plots for *S*. *italica*. For both species, our models suggest a higher seedling mortality during the first weeks in natural compared to amplified drought conditions in the undisturbed plots. This pattern then reversed after the 5th week for *S*. *italica* and after the 15th week for *S*. *nutans*, with seedling mortality becoming higher under amplified drought. We repeatedly found predation marks on cotyledons (missing fragment of leave and mucus from slugs) during the first weeks of the experiments. Therefore, we believe that higher seedling mortality during the autumn under natural drought could be explained by a greater predation pressure by herbivores favored by higher soil moisture. For example, activity and detectability of ground beetles and snails depend on soil humidity, and they tend to bury in deeper soil layers as it dries up (Hervé, 1959). During winter, colder temperatures constrain herbivore activity (Defossez et al., 2016), and this could explain the reversal in seedling mortality, increasing under the amplified drought treatment by the end of the winter.

As for seeding emergence, amplified drought had no effect on *S*. *italica* in disturbed plots. This may result from a more efficient rooting strategy and a higher water use efficiency of this species (O’Brien, Reynolds, et al., 2017). Indeed, the sensitivity to amplified drought may increase as the soil dries out, accompanied by an increase in water use efficiency. However, the wilting of the more resistant species to drought, here *S*. *italica* based on its distribution, would begin at a relatively lower value of soil water content compared to *S*. *nutans* (Belluau, 2017).

## 5 CONCLUSION

Our study investigated seedling emergence and seedling mortality, at a weekly time scale, and revealed strong negative effects of amplified drought. Yet, other vital rates, such as fecundity or adult plant mortality, may also be affected under climate change and contribute to overall population dynamics (Gimenez‐Benavides et al., 2008; Gómez‐Aparicio et al., 2008; Töpper et al., 2018). Hence, future studies should aim at simultaneously capturing more vital rates to refine the expected impacts of climate change on population dynamics.

We focused on the autumn and winter seedling dynamics, important in Mediterranean‐type ecosystems, which have not received much attention. Yet our results match that of earlier studies in the sense that amplified drought negatively impacts seedling emergence and increases seedling mortality. This suggests that amplified drought has consistently negative impact on seedling recruitment and drastic effects on population dynamics for a large number of species under many environmental conditions and in ecosystems.

## AUTHOR CONTRIBUTION

**Suzon Garnier:** Data curation (equal); Formal analysis (lead); Writing‐original draft (lead); Writing‐review & editing (equal). **Emma Giordanengo:** Data curation (equal); Formal analysis (equal); Writing‐original draft (lead); Writing‐review & editing (equal). **Arne Saatkamp:** Conceptualization (equal); Methodology (equal); Supervision (lead); Writing‐original draft (equal); Writing‐review & editing (equal). **Mathieu Santonja:** Conceptualization (equal); Methodology (equal); Writing‐review & editing (equal). **Jean‐Philippe Orts:** Data curation (lead); Methodology (equal); Writing‐review & editing (equal). **Ilja M. Reiter:** Conceptualization (equal); Data curation (equal); Writing‐review & editing (equal). **Thierry Gauquelin:** Conceptualization (equal); Writing‐review & editing (equal). **Eric Meineri:** Conceptualization (lead); Data curation (equal); Formal analysis (equal); Methodology (equal); Supervision (lead); Writing‐original draft (equal); Writing‐review & editing (lead).

## Data Availability

The data supporting this article is available from the Dryad Digital Repository: https://doi.org/10.5061/dryad.qrfj6q5h8.
